# Modular barcode beads for microfluidic single cell genomics

**DOI:** 10.1038/s41598-021-90255-x

**Published:** 2021-05-25

**Authors:** Cyrille L. Delley, Adam R. Abate

**Affiliations:** 1 Bioengineering and Therapeutic Sciences, University of California, San Francisco, CA 94158 USA; 2 California Institute for Quantitative Biosciences, University of California San Francisco, San Francisco, CA 94158 USA; 3 Chan Zuckerberg Biohub, San Francisco, CA 94158 USA

**Keywords:** High-throughput screening, Transcriptomics, Cancer genomics, Genomic analysis, Gene expression profiling

## Abstract

Barcode beads allow efficient nucleic acid tagging in single cell genomics. Current barcode designs, however, are fabricated with a particular application in mind. Repurposing to novel targets, or altering to add additional targets as information is obtained is possible but the result is suboptimal. Here, we describe a modular framework that simplifies generation of multifunctional beads and allows their easy extension to new targets.

## Introduction

Biological samples from tissues or the environment often contain communities of distinct cell types. Such mixtures can be deconvoluted a priori by isolating subsets for analysis, or a posteriori by employing methods which provide measurements at single cell resolution. For example, single cell genomics (scDNAseq) allows identification of the clonal composition of cancer cells, while single cell transcriptomics (scRNAseq) enables deconvolution of mixed cells with distinct phenotypes^1^. Because single cell approaches require little prior knowledge about the sample and because recent microfluidic methods provide enough throughput to characterize large and heterogeneous cell populations, these methods are becoming ubiquitous in biological research.

**Figure 1:**
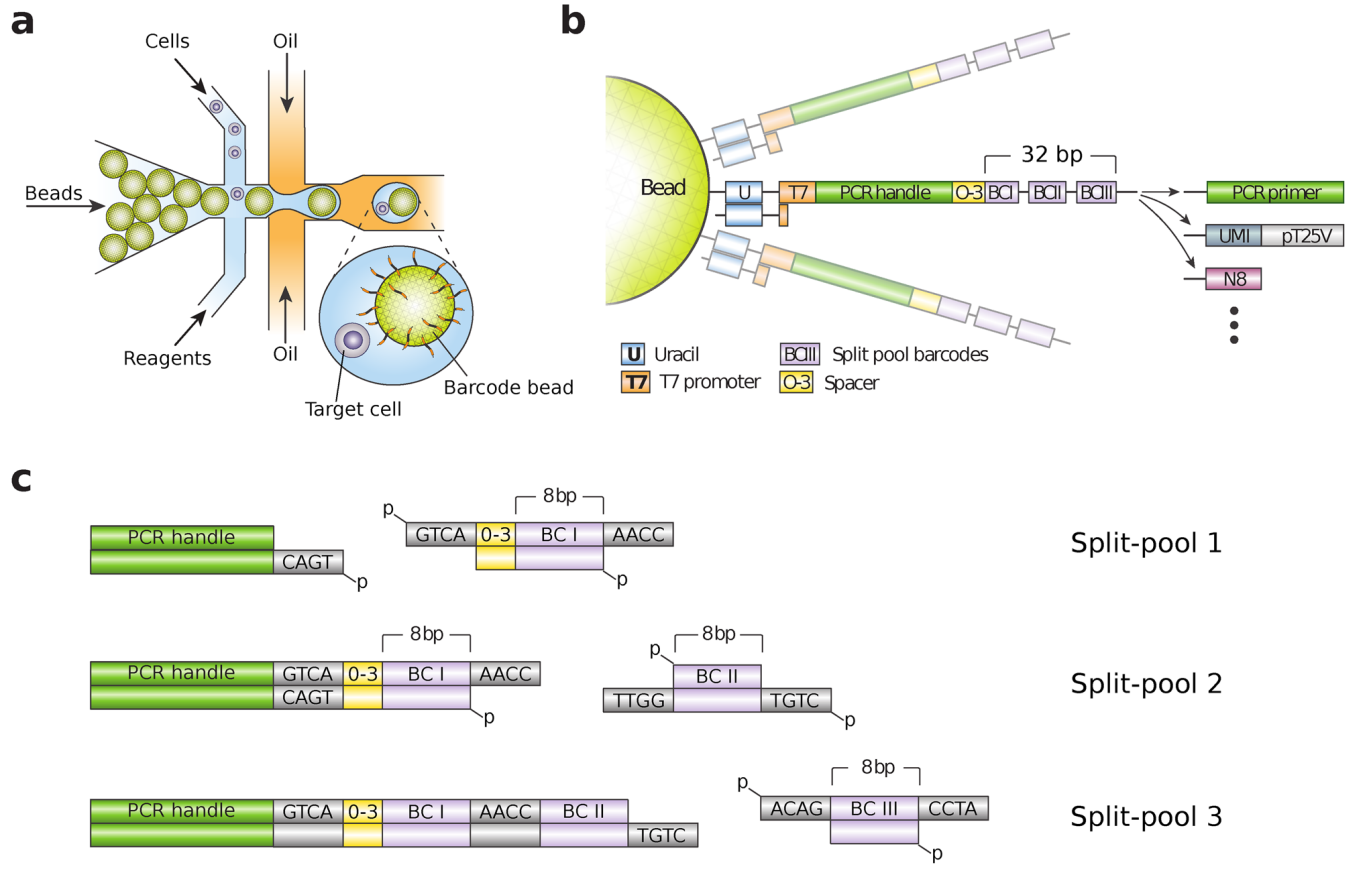
Barcode bead fabrication overview. (**a**) Barcode beads form the backbone of microfluidic single cell genomic protocols. Close packing enables efficient bead loading. (**b**) Organization of the barcode sequence on the beads. The elements are modular and can be removed or replaced with other sequence blocks if desired. The 0–3 bp spacer introduces a barcode specific frame shift that helps with cluster detection in Illumina’s sequence technolgoy. (**c**) DNA ligation with T4-ligase is used to minimize the footprint of the split-pool barcode fragments enabling more split pool cycles per length.

A key step in single cell genomics workflows is loading droplets with high concentrations of barcode oligonucleotides to tag nucleic acids of interest; the resultant tagged molecules can be pooled for all cells and efficiently sequenced in a single run. Barcode loading is often accomplished using beads on which barcode sequences are synthesized^2,3^ which provides two advantages: efficient microfluidic techniques allow most cells to be paired with a bead^4^ (Fig. 1a), while efficient synthesis of oligos on beads provides ample barcode for target tagging, yielding optimal sequencing data. However, current barcode designs utilize fixed targeting primers that are not easily repurposed; consequently, if new targets are identified, a new batch of beads must be synthesized, which is expensive and laborious. For example, repurposing beads used for scRNAseq to target genomic DNA results in barcodes containing poly-T stretches that prevent common sequencers from reading into downstream sequences; nor can additional targets be easily added to existing whole transcriptome or multiplexed amplicon beads, which would allow, for instance, sensitive capture of guide RNAs used in genome wide knockout screens^5,6^. If a universal barcode bead could be designed that could be easily and cost effectively retargeted to new sequences, it would allow easy repurposing of existing beads to new targets, accelerating single cell experiments and reducing cost and waste.

Here, we describe a modular framework that allows easy repurposing of existing bead batches to new targets (Fig. 1b,c) (“Supplementary Protocol”). The modular design is compatible with described single cell sequencing workflows, including scDNAseq^7^, scRNAseq^2^, Abseq^8^, multimodal analyses^9–12^, and genome wide knockout screens^13–15^. Moreover, the optimized structure generates compact barcodes that can be assembled in a fraction of the time and cost compared to existing methods. The platform is general and flexible, making it useful for numerous single cell genomics applications.

## Results

### Compact barcode synthesis by split-pool ligation

The core concept of our bead design is to use modular sequence assembly in the synthesis process. Unlike existing approaches which synthesize and target beads in one workflow, thereby yielding beads limited to one sequencing task, we design beads in which the barcode sequence is assembled in a first step, and appropriate primers as targeting moieties are added in a second step, such as poly-T for scRNAseq or multiplexed panels for scDNAseq.

Barcode bead fabrication starts with microfluidic emulsification of a gel precursor solution serving as the bead scaffold^16,17^. Once polymerized, we recover the beads and the covalently linked primers serve as anchors for barcode synthesis.

To synthesize barcode sequences on the beads, we use a split-pool approach. Split-pool assembly works by repeatedly partitioning the beads into random fractions, modifying the beads with a specific barcode fragment and pooling the beads. This results in a barcode set which grows exponentially with the number of cycles. Because the acrylamide hydrogel backbone of the beads is incompatible with phosphoramidites oligo synthesis we use enzymatic reactions to concatenate pre-synthesized oligos. As concatenated subsequences we use octamers from a library with minimum Levenshtein distance four^18,19^**,** which enables robust barcode identification even with sequencing errors and indels from oligo synthesis (Supplementary Data Tables 1–6).

DNA polymerases have previously been used in split-pool protocols, but require handles for specific hybridization, inflating barcode length. Barcodes can also be assembled with DNA ligases which can operate with four or less base pair overhangs^20–24^ (Supplementary Fig. 1a,b). These overhangs ensure specific ligation, since different sequences used in sequential steps prevent improper propagation of reactive stubs remaining from failed ligations in previous rounds. To characterize the process, we measure ligation efficiency in a split pool synthesis reaction, observing > 80% of stubs are ligated per round, such that after three rounds 64% of oligos on the bead are full length (Supplementary Fig. 1c). In contrast, beads fabricated using polymerases achieve just 36% yield after two rounds^17^. Thus, our results demonstrate that ligation is more efficient for barcode bead fabrication in split-pool protocols than polymerase extension while also yielding more compact barcodes that reduce sequencing waste.

A higher yield means that ligation can support the assembly of more barcode blocks compared to polymerase extension. We therefore assemble three blocks into a barcode while polymerase extension protocols use only two. The result is that 884,736 different sequences can be encoded from three 96-well plates of subsequences, which allows to profile 45,382 cells per experiment at a 5% clash rate. However, the high ligation efficiency would support adding a fourth block if a higher diversity becomes necessary. By contrast, using polymerase extension to assemble two-block barcodes from eight 96 well plates yields just 147,456 barcodes, allowing just 7,564 cells to be profiled. To match the same diversity in two rounds would require eighteen 96-well plates.

In terms of cost (Table 1), ligation is equivalent to polymerase extension per fabricated bead volume. However, the massive reduction in required barcode subsequences (three rather than 18) affords a significant lower upfront investment. Ligation is also faster and less laborious: it uses double stranded DNA while polymerases require single stranded, obviating the need to denature after each cycle and reducing the number of wash steps. Moreover, the considerably smaller number of oligos makes manual pipetting feasible, whereas synthesis of two-block polymerase libraries from 18 plates requires robotics to ensure quality beads.

**Table 1:** Bead fabrication cost overview: bead costs of InDrops beads are compared to the here described approach.

	InDrops gel beads	746496	Barcode space				
		Scale order	Yield guarantee	Set-up cost	Bead volume yield	Cost per ml bead	Cost per experiment (~ 20,000 cells)
1	InDrops Acrydite p	10,000	125 nmol	3218.8 $	5.7 ml	569.7 $/ml	28.48 $
18	Barcode Plates	10	– nmol	15,042.6 $	80.0 ml	188.0 $/ml	9.40 $
1	BST 2.0	8000	– units	283.0 $	8.6 ml	33.0 $/ml	1.65 $
1	dNTP	40	– umol	250.0 $	14.8 ml	16.9 $/ml	0.85 $
	Total			18,794.4 $		807.7 $/ml	40.38 $

	Ligation gel beads	884736	Barcode space				
		Scale order	Yield guarantee	Set-up cost	Bead volume yield	Cost per ml bead	Cost per experiment (~ 20 000 cells)
1	pBB1	10,000	1600 nmol	1738.4 $	72.3 ml	24.0 $/ml	1.20 $
3	Barcode Plates	25	– nmol	902.9 $	12.0 ml	75.2 $/ml	3.76 $
3	Splint Plates	100	– nmol	1199.5 $	50.0 ml	24.0 $/ml	1.20 $
1	pBB2 (phos-lig_start)	10,000	1600 nmol	1107.2 $	72.3 ml	15.3 $/ml	0.77 $
1	pBB3 (release-3C)	1000	100 nmol	102.0 $	16.0 ml	6.4 $/ml	0.32 $
1	pBB4 end splint	1000	200 nmol	21.0 $	10.7 ml	2.0 $/ml	0.10 $
1	pBB5 (phos-RNA)	1000	200 nmol	126.9 $	10.7 ml	11.9 $/ml	0.59 $
1	T4 PNK	2500	– units	228.0 $	13.0 ml	17.5 $/ml	0.88 $
1	T4 Ligase	100,000	– units	256.0 $	4.2 ml	61.4 $/ml	3.07 $
1	USER	250	– units	297.0 $	8.3 ml	35.6 $/ml	1.78 $
	Total			5978.9 $		273.4 $/ml	13.67 $

Costs are calculated based on list prices of vendors and are given as “set-up cost” that is the up front investment to start bead fabrication and as “cost per ml” reflecting the steady state cost per batch. Cost per experiment assumes a consumption of 50 μl beads. This amount allows profiling of about 20,000 cells; bead consumption for fewer cells will be similar because of dead volumes.

### Enzymatic barcode release is cost effective

Popular protocols for single cell genomics release barcodes from beads to increase availability for reverse transcription or PCR priming. This is normally achieved using UV cleavable chemical moieties, like 2-nitrobenzyl, or disulfides that can be broken with a reducing agent^2,25^. While fast to cleave, these linkers are expensive (Table 1) and require care to avoid premature cleavage during bead fabrication and handling. Our protocol instead employs enzymatic cleavage, yielding significant cost savings while also making the approach less sensitive to premature cleavage. Because genomic DNA is a valuable substrate in many single cell experiments, we avoid restriction enzymes including rare cutting Type IIS enzymes. Thus, we incorporate deoxyribose uracil (dU) as the linker, which does not exist in genomic DNA and can be cleaved by an enzyme mix comprising uracil DNA glycosylase and endonuclease III. Oligos containing dU are readily synthesized and inexpensive, and the linker consumes just one base. Moreover, dU containing oligos are stable and specifically cleavable by these enzymes, which are also readily available and inexpensive.

To characterize the efficiency of this barcode release strategy, we incubate beads functionalized with oligos containing dU in PCR and reverse transcription buffer, mimicking conditions of single cell genomics protocols. To measure cleavage efficiency, we use FAM-labelled probes complimentary to the cleavable oligo and measure fluorescence for cleaved beads and positive and negative controls. We observe little activity on single stranded oligos, but near complete cleavage of double stranded in both buffer conditions (Supplementary Fig. 1d–f). This makes sense because the enzymes natural substrate is double stranded DNA. Thus, to make the single stranded barcodes cleavable, we include an oligo complementary to the cleavage region in the mix, making it locally double stranded and achieving efficient cleavage. These results show that uracil cleavage is an efficient mechanism for barcode release, while reducing bead fabrication cost to about a third (Table 1).

### Flexible bead usage for scDNAseq and scRNAseq

**Figure 2:**
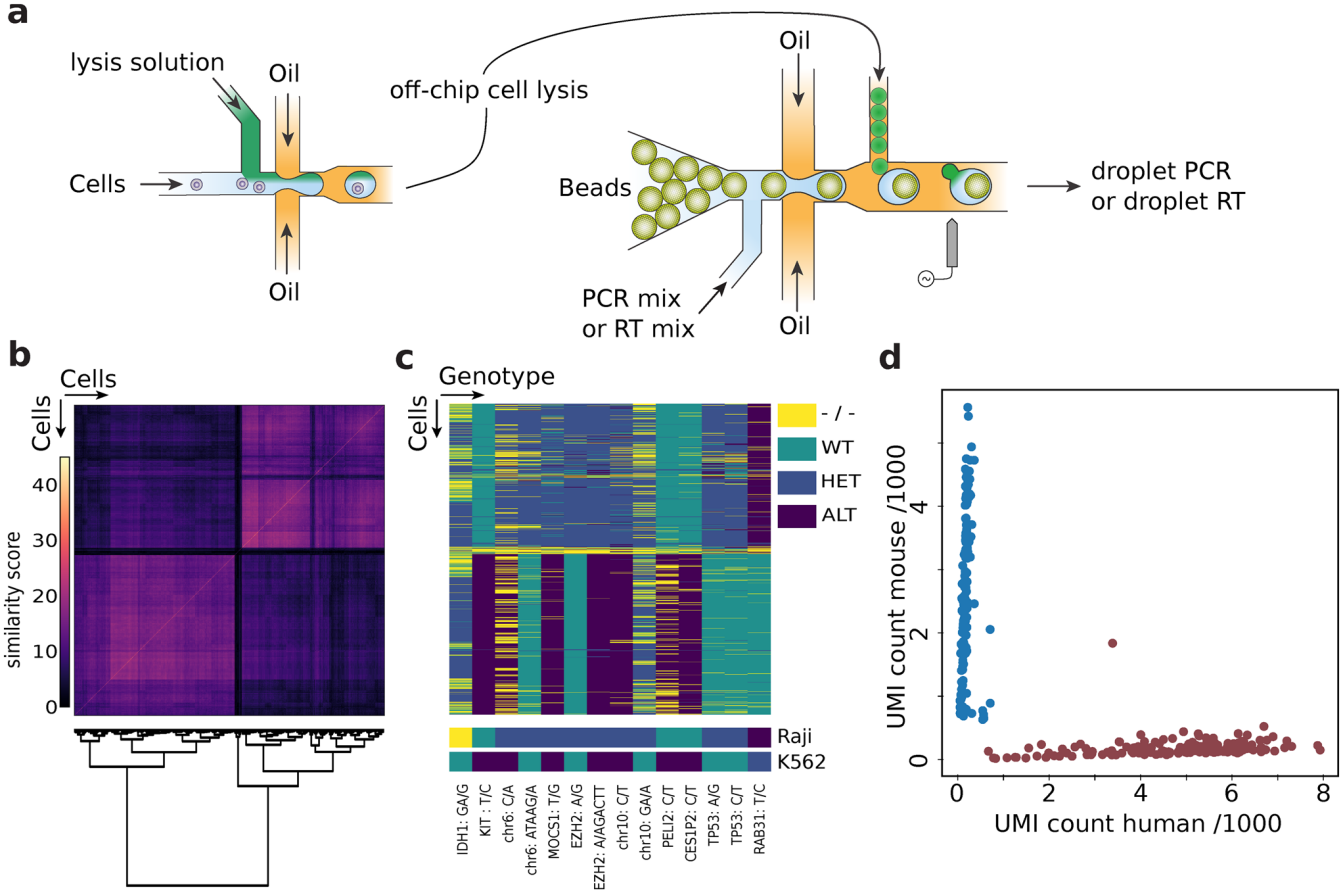
Singl-cell genotyping with barcode beads. (**a**) Schematic of the employed microfluidic two step protocol. A cell suspension is co-flowed with porteinase K or detergents and reinjected into a second device after off-chip incubation and heat inactivation of the protease. In the second device droplets with barcode beads are paired with cell lysate and PCR or RT reagents to create barcoded amplicons of targeted genomic regions. (**b**) Cell–cell similarity matrix based on the number of shared SNPs is given. Cells are ordered along both axes by hierarchical clustering using Ward’s minimum variance method. (**c**) Cells by genotype matrix, the cells (rows) are in the same order as in (**b**) and gnomic variants which are different between the two cell lines are given as columns. Genotyping dropouts are indicated in yellow. Bottom rows shows genomic variants detected from homogeneous bulk samples (full variant indexes are given in “Supplementary Data Table”). (**d**) Single cell RNA sequencing result of the Mouse (3T3) Human (K562) species mixing experiment, counts represent detected unique molecular identifiers in thousands.

To demonstrate the benefit of the modular bead design in typical use cases we create a mock cancer cell system consisting of two human cell lines, Raji and K562, and profile their clonal relationship. We exploit that many cancers are driven by recurring mutations at hot spots in tumour suppressors and proto-oncogenes. To infer clonal relation between cells it is therefore beneficial to sequence these hot spots only rather than the full 6.2 mega-basepairs of the (diploid) human genome. This ensures high coverage of the most relevant genomic areas at two to three orders of magnitude lower cost. We select a panel of 49 genomic locations covering about 12,500 basepairs (Supplementary Data Table 8) that are known hot spots for mutations in acute myeloid leukemia, functionalizing the beads with the corresponding set of 49 forward primers, and running the associated microfluidic workflow^7^. After cell encapsulation, lysis, and chromatin digestion, we merge the droplets with barcode beads, PCR reagents, and enzymes to cleave the uracil linkers, thereby releasing the barcode primers into solution for targeted amplification of the 49 loci (Fig. 2a). The resultant sequence data yields high quality reads across the panel for 1020 cells, with a median of 3447 reads and 45 detected amplicon loci per cell (Supplementary Fig. 2a,b).

After aligning the fragments to the human reference genome and performing variant calling, we use hierarchical clustering to assign genotypes via Ward's minimum variance method^26^**,** obtaining two separated clusters (Fig. 2b). A comparison of the detected variant calls in single cells with the genotype obtained from a bulk measurement of Raji and K562 cells shows that the clusters indeed represent the two input lines (Fig. 2c) (Supplementary Data Table 9). We apply the same workflow to other cell lines such as P493-6, LAX7R; and profile CEM and K562 with the same beads but a different microfluidic strategy^27^, to demonstrate that the method is insensitive to cellular differences and can be used in different microfluidc settings. In all cases we obtain single cell libraries that allow distinguishing the cell lines based on genomic polymorphisms (Supplementary Figs. 2c–f and 3a,b). We note however that the smaller panel run on the alternate microfluidic approach exhibits a higher fraction of amplicon dropout (Supplementary Fig. 2d,f); and the relative performance of individual amplicons is changed (Supplementary Fig. 2e,c). These experiments show that our barcode beads enable high throughput single cell genome sequencing with data quality equivalent to traditional beads.

A common quality control experiment in single cell RNA sequencing is to profile a cell suspension of two species because individual transcripts can be assigned to cell type based on their sequence. To showcase the potential of the modular bead design for scRNAseq we therefore attach a poly-T primer instead of the cancer hot spot panel to the same bead batch and prepare a mixed mouse (NIH 3T3) and human (K562) cell sample. We use the same microfluidic workflow, but include an acidic lysis solution to stabilize RNA, and reverse transcription (RT) instead of PCR reagents, to produce a scRNAseq library. We collect droplets for ~ 1 min aiming to capture 300–400 cells; sequencing yields 164 mouse cells with a median count of 2688 transcripts, 189 human cells with 5330 transcripts (Fig. 2d). This demonstrates that our repurposed beads perform successfully in scRNAseq experiments (Supplementary Fig. 4a–f)^2^. Bead modularity thus enables rapid deployment of the same barcode beads for different single cell workflows targeting distinct molecules.

## Discussion

Our modular barcode design reduces the cost of bead synthesis while allowing rapid and inexpensive repurposing to other targets, including DNA and RNA. This makes them attractive for multiomic experiments by allowing fine tuning of primer sequences and concentrations to obtain best conditions for simultaneous DNA and RNA sequencing. This is important, because research on designing primers for multiplexed PCR reaction demonstrates that countering primer bias is a non trivial problem which requires empirical optimization to achieve good results^28–31^. It is therefore possible that the reduced efficiency in our 16 amplicon experiment is caused by the removal of primers from the full panel. Since primers compete for a shared resource in a multiplexed PCR, removing competitors can perturb the stability of the system. These effects highlight the value of a modular bead design that allows quick tuning of primer composition and concentration to optimize these experiments.

Moreover, the barcode sequences are compact and efficiently synthesized to full length, reducing sequencing waste and minimally consuming read length. A compact barcode structure also allows to use PCR primers to amplify specific cells of interest to increase their sequence coverage and improve signal quality in the pooled library^32^. The ease with which highly multiplexed primers can be added enables new opportunities to enhance scRNAseq sensitivity. For example, poly-T mRNA capture yields an unbiased profile of expressed transcripts, but because coverage is typically below ~ 10%, low abundance transcripts may be missed. Our modular design might be used to enhance detection of such transcripts by dedicating a fraction of all primers on the bead to their capture alongside poly-T probes. Besides established DNA or RNA protocols, we expect that bead modularity will accelerate the introduction of novel assays by reducing the risk of fabricating dedicated bead batches that may not work.

## Methods

### Microfluidic device fabrication

Devices were fabricated with standard photolithography techniques^33^. Custom device fabrication is not necessary to use these beads and can be substituted with commercially available instruments (e.g. from 10 × Genomics, Mission Bio, 1CellBio and others). Master structures were made with Su8 3025 photoresist (MicroChem, Westborough, MA, USA) on a three inch silicon waver (University Wafer) by spin coating, soft baking at 95 °C for 20 min and subjecting to 3 min UV-exposure through printed photolithography masks (CAD/Art Services, 12,000 DPI) (Supplementary File 2). Post UV exposure, the wafer was baked at 95 °C for 2 min and cooled to room temperature and developed in a propylene glycol monomethyl ether acetate (Sigma Aldrich) bath, rinsed with acetate, and dried and hard baked at 225 °C for 10 min. Curing agent and PDMS were mixed in 1:10 ratio, degassed and poured over the master structure and baked at 65 °C for 4 h, removed from the master and punched with a 0.75 mm biopsy core (World Precision Instruments). The device was then bonded to a glass slide using O_2_ plasma and the channels were treated with Aquapel (PPG Industries) to render them hydrophobic. Aquapel was purged from the channels with air after 5 min contact time and residual liquid evaporated by baking at 65 °C for 15 min.

### Barcode bead synthesis

Barcode beads were prepared by generating droplets on a microfluidic drop maker (Supplementary Fig. 5) with an acrylamide premix (6% w/v Acrylamide, 0.15% w/v N,N′-Methylenebisacrylamide, 48 mM Tris–HCl pH 8.0, 0.3% w/v ammonium persulfate, 0.1 × Tris-buffered saline–EDTA (TBSET: 10 mM Tris–HCL pH 8.0, 137 mM NaCl, 20 mM EDTA, 1.4 mM KCl, 0.1% v/v Triton-X100), 20 mM primer pBB1 (Supplementary Data Table 7): /5Acryd/ACTAACAATAAGCTCUAUCGATGACCTAATACGACTCACTATAGGGACAAATGCC GATTCCTGCTGAAC (IDT) as dispersed phase and HFE-7500 (3 M Novec) with 2% (w/v) PEG-PFPE amphiphilic block copolymer surfactant (008-Fluoro-surfactant, Ran Technologies) and 0.4% v/v Tetramethylethylenediamine as continuous phase. The resulting emulsion was kept at room temperature over night to let the acrylamide polymerize. The emulsion was broken with 1H,1H,2H,2H-Perfluoro-1-octanol (Sigma Aldrich) and the beads washed three times in TBEST, three times in Tris–EDTA-Tween buffer (TET: 10 mM Tris–HCl pH 8.0, 10 mM EDTA, 0.1% v/v Tween-20), and three times in pre-ligation buffer (30 mM NaCl, 10 mM Tris–HCl pH 8.0, 1 mM MgCl_2_, 0.1% Tween-20). Beads were resuspended in T4 ligation buffer (NEB) and 7.5 μM of primer pBB2: /5Phos/TGACGTTCAGCAGGAATCGGCATTTGTCCCTATAGT GAGTCGTATTAGGTCATCGATAGAG at approximately 1:1 solid:solvent fraction. The suspension was heated to 75 °C and slowly cooled to room temperature to anneal the primer pair.

Barcode primer plate and splint plate (Supplementary Data Tables 1–6) for first round of split-pool barcode synthesis was prepared by combining each barcode with its cognate splint at a 1:1 ratio and a final concentration of 100 μM. Paired barcode and splints were phosphorylated in a PCR plate by combining 20 μl of oligonucleotides with 40 μl phosphorylation mix (1× T4 ligation buffer, 0.2 mg/ml BSA, 0.167 U/μl T4 PNK (NEB)) and incubated for 30 min at 37 °C and heat inactivated for 20 min at 65 °C. After phosphorylation, 100 μl of bead suspension to each barcode splint pair. To start the first round of ligation 40 μl of ligation mix (9.54 U/μl T4 ligase (NEB) in 1 × T4 ligase buffer) was added to each well and the plate sealed and incubated for 1–4 h at room temperature and the enzyme inactivated by heating to 65 °C for 10 min. Beads were collected, washed five times in TET and resuspended in ligation buffer at a 1:1 solid:solvent fraction for the next round of ligation. This process was repeated for the barcode fragments 2 and 3 to yield the final modular gel beads which can be quickly functionalized for a specific purpose.

To prepare the barcoded beads for single cell experiments we resuspended the beads at 1:2 solid to liquid in T4 ligation buffer and performed a splinted DNA ligation for 1 h at room temperature. For RNA beads we used 10 μM pBB4: CTCGAATAGG as splint and 10 μM pBB5:/5Phos/TTCGAGNNNNNNNNTTTTTTTTTTTTTTTTTTTTTTTTV as primer. For the cancer hot spot panel we phosphorylated a set of 49 primers (Mission Bio Acute Myeloid Leukemia Panel) or 16 primers (Supplementary Data Table 8) at equimolar ratio and ligated to the beads at 10 μM total concentration using 10 μM pBB8: CTGCGAGTACTAGG or pBB4 as splint. To render the barcodes single stranded, beads were washed four times in denaturing solution (100 mM NaOH, 0.5% v/v Brij-35) and washing solution quenched by resuspending in low salt buffer (10 mM NaCl, 10 mM Tris–HCl pH 8.0, 0.1 mM EDTA, 0.1% Tween-20).

A detailed step-by-step description is provided as “Supplementary Protocol”. The protocol yields 2.5 ml or about 25 million beads which lasts for about 50 single cell experiments that can profile 20,000 cells each. Experiments profiling fewer cells will still consume 20–50 μl of beads due to dead volumes in the microfluidic setup.

### Barcode release test

Barcode beads (2 μl) were resuspended in LS (10 mM Tris–HCl pH 7.5, 1 mM MgCl2, 50 mM NaCl, 0.1% Tween20) and incubated for 1 min. The beads were pelleted at 2000 g, the supernatant removed and the beads resusbended in 20 μl 1 × CutSmart (New England Biolabs), 1 × Maxima H- RT buffer (Thermo Scientific) or 1 × Kappa HiFi PCR buffer (Kapa Biosystems). For locally double-stranded cleavage tests pBB3 was added to a final concentration of 3 μM. Then, 0.4 μl USER II (New England Biolabs) enzyme mix was added an the suspension incubated for 45 min at 37 °C, and heat inactivated. FAM-probe pBB6 was added to a final concentration of 1 μM and the beads incubate at room temperature for 15 min under rotation. Beads were washed three times in 1 ml TET and imaged on a fluorescence microscope (EVOS cell imaging systems, Thermo Fisher) at constant light intensity, shutter speed and signal amplification.

For bioanalyzer traces 1 μl beads were resuspended in 20 μl CutSmart and released with 0.5 μl USER II by incubating 30 min at 37 °C. The Samples were diluted fourfold with water and 1 μl supernatant was loaded on a Bioanalyzer High Sensitivity DNA electrophoresis chip (Agilent Technologies).

### Cell culture

K562 (ATCC CCL-243); Raji (ATCC CCL-86), CCRF-CEM (ATCC CCL-119) and P493-6 (Cellosaurus CVCL_6783); NIH 3T3 (ATCC CRL-1658); or LAX7R^34^ (gift from Jim Wells laboratory) cells were cultured at 37 °C in the presence of 5% CO2 in Iscove's Modified Dulbecco's Medium; RPMI-1640 medium; Dulbecco's Modified Eagle's Medium; or MEMα with l-glutamine & Ribo- & Deoxyribonucleosides. Each media was supplemented with antibiotics and 10% fetal bovine serum (FBS). Human cell lines were washed in PBS, 3T3 were washed in PBS and detached by trypsinization to form a cell suspension for microfluidic experiments.

### Equipment for microfluidic operation

Syringe pumps (NE-500 Programmable OEM Syringe Pump, New Era Pump Systems) were controlled with a custom python script (see data availability) and the operation monitored on an inverted microscope. The syringes were connected to the microfluidic device with PE tubing (Scicominc, #BB31695-PE/2). To generate an electric filed for droplet merging a DC power supply (HY3003D) was used and set to 1.6 V output. DC current was converted to AC with a Elevam P-878 power inverter. Droplet merging was achieved using 1 M NaCl as electrode^35^.

### Cell encapsulation and barcoding

For the cancer hot spot experiment K562 and Raji cells or P493-6 and LAX7R cells were mixed at a 1:1 ratio and resuspended in phosphate-buffered saline (PBS) with 10.2% (w/v) Iodixanol at an approximate concentration of 3 million cells/ml. Cells were co-flowed with lysis buffer (100 mM Tris at pH 8.0, 0.5% IGEPAL, proteinase K 1.0 mg/ml)^36^ each at 1500 μl/h and with 3000 μl/h HFE-7500 with 2% (w/v) PEG-PFPE amphiphilic block copolymer on a bubble-trigger device^37^ (Supplementary File 2) to form droplets of about 45 μm diameter. Droplets were collected and incubated at 50 °C for 1 h and 80 °C 10 min to lyse the cells and heat inactivate proteinase K. On a bead-and-droplet merging device (Supplementary Fig. 5) closed packed, hot spot panel modified barcoding beads in DNA bead buffer (10 mM Tris–HCl pH 7.5, 40 mM NaCl, 2.5 mM MgCl_2_, 3.75% (v/v) Tween-20, 2.5% (v/v) Glycerol, 0.625 mg/ml BSA, 3 μM pBB3: TCATCGATAGAGCTTATTGT/3C6/) were reinjected at 75 μl/h and co-flowed with 150 μl/h PCR mix (1.65 × NEBNext Ultra II Q5 Master Mix, 0.033 U/μl USER II (NEB), 1.32 M Propylene glycol, 0.25 mg/ml BSA, 0.5 mM DTT) to form bead containing droplets which were merged with the cell lysate containing droplets reinjected at 35 μl/h (about 200 Hz) by a salt water electrode^34^ (Fig. 2a). The droplets equivalent of about 1000 cells (69 s fractions) were collected, the oil excess oil removed from the collection tubes and replaced with FC40 with 5% (w/v) PEG-PFPE amphiphilic block copolymer. Droplet PCR was thermocycled with the following conditions: 30 min at 30 °C to release primers, 3 min at 95 °C; 20 cycles of 20 s at 98 °C, 10 s at 72 °C, 4 min at 62 °C, and 30 s at 72 °C; and a final step of 2 min at 72 °C with all ramp rates set to 1 °C/s. Emulsion was broken with 1H,1H,2H,2H-Perfluoro-1-octanol diluted with 60 μl water the beads pelleted and 50 μl supernatant removed. To supernatant 5 μl 10 × CutSmart (NEB) was added and incubated with 20 units ExoI nuclease (NEB) for 1 h at 37 °C before purification with 42 μl AMPure beads (Beckman Coulter). The sample was eluted in 20 μl water which served as input for sequencing library generation. Cancer hotspot experiments with CCRF-CEM and K562 cells were done as previously described^27^.

For the RNA experiment K562 and 3T3 cells were mixed at a 1:1 ratio and resuspended at 2.57 million cells/ml in the same buffer as above. Cells were co-flowed with lysis buffer (30 mM Na-citrate pH 6.5, 0.2% Trition-X100, 0.2% SDS, 2 mM EDTA, 10 mM DTT) on the same device as above, droplets collected and incubate 1 h at 4 °C. Same bead-and-droplet merging device was used to combine pBB5 functionalized, closed packed barcoding beads in RNA bead buffer (1 × Maxima H- RT Buffer (ThermoFisher), 2% (v/v) Tween-20, 0.625 mg/ml BSA, 3 μM pBB3). Beads were reinjected at 75 μl/h and co-flowed with 150 μl/h RT mix (1.65 × Maxima H- RT Buffer, 0.033 U/μl USER II (NEB), 15 U/μl Maxima H- RT (ThermoFisher), 1.65 U/μl RNasin (Promega), 1.65 mM dNTP (NEB), 2.2% (v/v) Tween-20) and merged with the cell lysate containing droplets reinjected at 25 μl/h (about 200 Hz) same as above. The droplets equivalent of about 300–400 cells (60 s fractions) were collected and the oil exchanged as above. Emulsion was incubated at 37 °C for 30 min then 54 °C for 1 h. Emulsion was overlaid with 20 μl 1 × CutSmart containing 20 units of ExoI, broken with 5 μl 1H,1H,2H,2H-Perfluoro-1-octanol, and incubated for 30 min at 37 °C. RNA/DNA hybrids were purified with 40 μl AMPure beads and eluted in 20 μl water and stored at -20 °C.

### Library preparation and sequencing

To the cancer hot spot library P5 and P7 sequences were attached by PCR using the custom pBB9 primer and Nextera N701 (Illumina). Library was purified with 0.6 × volume fraction AMPure beads, its concentration measured by a fluorometer (Qubit 3.0, Invitrogen) and the absence of primer dimers verified on a Bioanalyzer High Sensitivity DNA electrophoresis chip (Agilent Technologies). For sequencing a MiSeq V2 300 cycles kit (Illumina) was used and the library diluted to 12 pM according to the recommendations of the sequencing kit manual. The library was sequenced in paired end mode and each side sequenced over 150 base pairs.

For RNA library, second strand synthesis and linear amplification by in vitro transcription (IVT) was done as described previously^17^ without fragmenting RNA after IVT. IVT product was reverse transcribed with the primer: AAGCAGTGGTATCAACGCAGAGTGTANNNGGNNNB^38^ as described^17^, purified wit 0.9 × volume fraction AMPure beads and the concentration measured by the Qubid dsDNA HS assay (Thermofisher). 13.8 ng RNA/DNA hybrid was fragmented with 20 μg Tn5^39,40^ (assembled with pBB11 and pBB12) in 50 μl. Library was amplified with pBB9 and pBB13 and NEBNext Ultra II Q5 according to the manufacturer protocol, purified with 0.65 × AMPure beads and prepared a 12 pM sequencing library. The library was sequenced on a MiSeq V3 150 cycles kit (Illumina) in paired end mode, distributed 55/110 cycles, using custom sequencing primers pBB10 and pBB11 (Supplementary Data Table 7).

### Bioinformatic data evaluation

Bead barcodes were parsed from the read 1 file with a custom script (see data availability). The program cutadapt (v2.4)^41^ was used to find the common ligation scar between the combinatorial barcodes and the forward read primers. This step is necessary because the 0–3 base pair spacer in the barcode bead sequence (which helps cluster identification on the sequencing device by increasing sequence diversity for otherwise identical segments of the barcode sequence such as the ligation scars) prevents extraction of the combinatorial barcode blocks by distance from the 5′-end. Barcode blocks are extracted by distance from the position of the ligation scar (3′-side distance) and matched against a white list, allowing a maximum Levenshtein distance of one, to identify true barcode sequences.

Read 1 and read 2 sequences were demultiplexed into barcode groups and valid cell barcode groups were discriminated from background barcode groups by identifying the inflection point of the barcode rank plot versus number of associated reads (the “knee method”). Reads from valid cell barcodes were processed as previously described^12^. Briefly, FASTQ files with valid reads were aligned to the hg19 build of the human genome reference using bowtie2 (v2.3.4.1), filtered (properly mapped, mapping quality > 2, primary alignment), sorted, and indexed with samtools (v1.8). HaplotypeCaller from the GATK suite (v.4.1.3.0) was used to produce GVCF files and genotyped jointly on all genomic intervals with GATK GenotypeGVCFs. Genotyped intervals were combined into a single variant call format (VCF) file and multiallelic records split and left-aligned using bcftools (v1.9). Finally, variant records were exported to HDF5 format using a condensed representation of the genotyping calls (0: wildtype; 1: heterozygous alternate; 2: homozygous alternate; 3: no call). The result is a cell by allelic variant matrix, **V**, with the condensed genotype call as categorial matrix elements.

To cluster cells, the allelic variant matrix was one-hot encoded, converting **V** to four binary matrices, **V**_**0**_ to **V**_**3**_. Pairwise cell–cell similarity was calculated by computing the dot product for the matrices corresponding to heterozygous and alternate calls and summing them: **V**_**1**_**V**_**1**_^**T**^** + V**_**2**_**V**_**2**_^**T**^, yielding the cell–cell similarity matrix **S**. Hierarchical clustering of **S** was done with SciPy (v1.3.1) using scipy.cluster.hierarchy.linkage and Ward’s minimum variance method. Variant calls that distinguish the two cell lines were identified by discarding all calls for which less than 10% of cells were called as alternate (sum of heterozygous and homozygous alternate) yielding 27 potentially informative variants. Remaining variant calls were inspected and discarded if constant over all cells. The remaining 14 variant call positions are the final list.

The fastq files from the single cell RNA sequencing experiment were preprocessed with a custom script (see data availability) to remove the 0–3 bp variable spacer, concatenate the coding portion of each barcode read and to call valid cell barcodes by the “knee method”. Thus prepared files were converted to a cell by gene count matrix with Kallisto^42^ (v0.46.2) and Bustool^43^. These tools were run with default parameters unless otherwise stated. The reads were demultiplexed into barcode groups and pseudoaligned to the human (hg19) and mouse (GRCm38) reference transcriptomes with the Kallisto bus command with option—× 0,0,24:0,24,32:1,0,0 (this specifies the first 24 bp of the read 1 file as barcode and the following eight as UMI. Read 2 is aligned to the reference transcriptomes). About 12 million reads out of 22 million aligned successfully. Pseudoaligned reads were converted to gene counts with Bustool. Upon data inspection cells where further filtered by requiring at least 775 UMI counts and no more than 5% mitochondrial reads (Supplementary Fig. 5b); no genes were filtered.This final cell by gene count matrix was visualized in python using Scanpy^44^ and matplotlib^45^.

## Supplementary Information

Supplementary Legends.

Supplementary Information 1.

Supplementary Data Tables.

Supplementary Information 2.

## Data Availability

All scripts are available on GitHub at https://github.com/AbateLab/ModularGelBeads, and https://github.com/AbateLab/Pump-Control-Program. All sequencing data generated in this study is available on the Sequence Read Archive under BioProject number PRJNA632423 and PRJNA660010 upon final publication (referee access through: https://dataview.ncbi.nlm.nih.gov/object/PRJNA632423?reviewer=ism950i1v42eqa1r77nep1aq0 and https://dataview.ncbi.nlm.nih.gov/object/PRJNA660010?reviewer=qm6uo40vv3cfdgfnl5gf22alvj).
