# Supplementary material for: Modular barcode beads for microfluidic single cell genomics
Source: Sci Rep. 2021 May 25;11:10857. doi: 10.1038/s41598-021-90255-x (PMC8149635; doi:10.1038/s41598-021-90255-x)
Supplement: Supplementary file 1 — Supplementary Legends. [file 41598_2021_90255_MOESM1_ESM.docx]

Supplementary Materials

Supplementary file 1: Bead fabrication protocol, supplementary figures and table

Supplementary data tables: Primer sequences, amplicon positions and variant calls

Supplementary file 2: microfluidic devices
